# Effective regulation of technology in women’s health and healthcare

**DOI:** 10.1136/bmj-2025-086300

**Published:** 2025-10-10

**Authors:** Sara Raza, Sara Gerke, Eric Bressman, Carmel Shachar

**Affiliations:** 1 Center for Health Law and Policy Innovation of Harvard Law School Cambridge, MA, USA; 2 University of Illinois Urbana-Champaign, College of Law and European Union Center, Champaign, IL, USA; 3 University of Pennsylvania Perelman School of Medicine, Philadelphia, PA, USA

## Abstract

**Carmel Shachar and colleagues** argue that femtech requires robust and stringent privacy and security safeguards because of the sensitivity of the data

The rise of direct-to-consumer technologies has produced rapid expansion of women’s health innovation in a sector popularly known as femtech. Femtech encompasses a “range of technology-enabled, consumer-centric products and solutions” targeted at female health needs,^1^ including maternal health, menstrual health, pelvic and sexual health, menopause, contraception, and many other health conditions that disproportionately affect women.^2^

One example highlighting the promise of femtech is an at-home cervical cancer screening tool that showed comparable effectiveness to traditional clinic based testing.^3^ Use of the tool could bridge screening gaps and improve health outcomes for women. Other examples of femtech include wearables and mobile applications such as period and fertility trackers that monitor menstrual cycles, biometric data, ovulation, and related reproductive and sexual health data.^4^

The femtech industry has grown tremendously, reaching an estimated value of $60bn (£44bn; €51bn) in 2024^5^ and projected to reach $103bn by 2030.^6^ This expansion reflects both unmet health needs and rising awareness about female specific conditions, but it also shows the urgent need for thoughtful regulation to ensure transparency, safety, and accountability. Social undercurrents and the broader political climate can make women’s health data (particularly reproductive or sexual health information) more vulnerable than other health data. Although femtech may not require separate regulation from other digital health technologies, heightened privacy and security protections are urgently needed for femtech data as well as steps to mitigate bias.

Femtech is used worldwide, but access and experience differ for women in the global north and south. This article, part of the BMJ Collection on Women’s Health Innovation (www.bmj.com/collections/womens-health-innovation), focuses on the global north, partly because these jurisdictions have been leaders in the regulation of health tech and AI and have an outsized influence on the products available across the world. We draw lessons primarily from the United States (US) and European Union (EU) to provide actionable next steps for regulators across the world, including those in the global south, with the caveat that not all of these recommendations might apply to other settings.

## Femtech raises unique legal and regulatory concerns

Since the US Supreme Court overturned the right to abortion established in Roe *v* Wade, the shifting landscape of reproductive health in the US has brought increased attention to femtech, particularly to period and fertility trackers.^7^ The Biden administration sought regulatory and administrative ways to protect reproductive data, including directing the Federal Trade Commission (FTC) to prosecute several femtech companies and issuing a rule under the Health Insurance Portability and Accountability Act (HIPAA) to better protect the data of patients seeking lawful abortion care.^8^

In the EU, Poland has one of the most restrictive abortion laws in Europe, despite growing advocacy to legalise abortion and protect women’s health and wellbeing.^9^ In 2022, Poland’s minister of health issued an ordinance establishing a pregnancy registry, requiring doctors to report each patient’s pregnancy to a national database.^10^ Although the registry was supposed to be accessible to only medical staff, it created a chilling effect and raised concerns about the potential for disclosure in family civil cases and state prosecutor investigations.^11^

Femtech often stores reproductive data and could therefore be used to prosecute women seeking certain types of reproductive care, including abortions, unless there are adequate privacy protections. While femtech apps may not inherently require separate regulation from other digital health products, the sensitive nature of the data they process, combined with the current social climate in many countries, makes them more vulnerable to scrutiny and surveillance (box 1). It is therefore essential that they incorporate strong data protection measures and meaningful user control over personal health data.

Box 1 **Case study (Glow settlement)**

In 2020, California attorney general, Xavier Becerra, announced a landmark settlement against Upward Labs Holdings and Glow for their mobile application (Glow app) that had serious basic privacy and security failures.^12^ The complaint noted that the “Glow app collect[ed] and store[d] deeply-sensitive personal and medical information related to a user’s menstruation, sexual activity, and fertility” and tracked several types of personal and medical information, from history of previous pregnancies to physical and emotional conditions, such as bloating, sore breasts, or sex drive.^12^ The Glow app also allowed users to import a complete medical record from another healthcare provider, as well as export information into a file that the user can take to their doctor’s appointment.

Security failures and activities from 2013 to 2016 that triggered violations of multiple laws, including California’s Confidentiality of Medical Information Act (CMIA)^12^ ^13^:

*Glow App’s partner connect feature—*This allowed Glow users to link to a partner to share information by automatically granting a partner’s link request and immediately sharing the user’s sensitive information, such as sexual activity. The complaint alleged that by automatically granting the link request and sharing sensitive user information, the companies failed to obtain any authorisation from the user before disclosing their medical information and failed to verify the legitimacy of the person with whom the information was being shared.
*Glow App’s password change vulnerability* **—**Glow users were allowed to request a new password by entering an old password that may not have necessarily matched their old password, resulting in new passwords always being accepted and anyone being able to change a user’s password and accessing their data.
*Glow’s privacy policy and terms of use* **:** The privacy policies and terms of use, which contained claims about how the companies protect consumer privacy and users’ personal information, contradicted Glow’s actual practices (eg, “[W]e have designed the Service to protect information about you from unauthorized disclosure to others.”; “We use industry standard security measures to protect your information so that it is not made available to unauthorized parties.”).^12^

This settlement imposed a civil penalty of $250 000, included injunctive terms that required Glow to comply with state consumer protection and privacy laws, and “a first-ever injunctive term that required Glow to consider how privacy and security lapses may uniquely impact women.”^14^ The injunctive terms also required Glow to incorporate privacy and security design principles into its mobile apps. Glow was also required to obtain affirmative consent from users before disclosing personal, medical, or sensitive information, and allow users to revoke previously granted consent.

The complaint also alleged that Glow is a “provider of healthcare” for the purposes of CMIA, which opens the door to other femtech software and devices being treated the same way under similar health data privacy laws.^13^ This landmark settlement indicates the breadth of serious risks that the rapidly growing femtech industry poses with its uniquely sensitive nature.

### Data privacy

Lack of visibility surrounding how femtech apps collect and store sensitive data prevents users from making informed choices about their personal health information. For example, an important privacy and transparency challenge in period and fertility trackers is data location (where the data are processed and stored), which may differ from the user’s physical location and be subject to different levels of data protection laws.^15^ How femtech data are regulated depends on each jurisdiction’s privacy law frameworks.

The US currently has no comprehensive federal privacy law that governs femtech data. The primary health data privacy law is HIPAA, which applies only to certain “protected” health information, typically found in electronic health records generated by “covered entities.”^16^ Femtech companies are not usually considered HIPAA covered entities, although they may qualify as a business associate of a HIPAA covered entity— for example, if they collect health information on behalf of a hospital.^17^

The US FTC has regulatory tools to police the use of data by femtech and other digital health products, but enforcement is usually limited to serious cases (table 1). For example, in 2021, the FTC invoked section 5 of the FTC Act to penalise the fertility tracking app Flo Health for disclosing sensitive health data to marketing and analytic firms.^18^ This action was taken under the FTC’s authority to police “unfair or deceptive practices,” which includes instances when an organisation violates its own privacy policies and breaches consumer trust.^19^

**Table 1:** Key legal and regulatory differences between data privacy protections and femtech regulation in the US and EU

	United States	European Union
**Femtech**	No comprehensive federal privacy law that governs direct-to-consumer (DTC) technologies such as femtech products and applications	The General Data Protection Regulation (GDPR) is comprehensive and consistent in scope
	The primary health data privacy law in the US, the Health Insurance Portability and Accountability Act (HIPAA), only covers certain protected health information that is typically found in electronic health records	Although the GDPR does not protect femtech data in particular, it has protections for personal data, and heightened protection for special categories of personal data, such as data concerning health or data on a person’s sex life or sexual orientation
	Some states have enacted health data privacy laws	“Data concerning health,” as defined under article 4(15), refers to “personal data related to the physical or mental health of a natural person, including the provision of health care services, which reveal information about his or her health status”
**Apps for children**	Children’s Online Privacy Protection Act (COPPA) is the primary federal law that protects children’s data privacy in the US	GDPR article 8(1) also protects children’s data privacy regarding information society services by only allowing processing of personal data of a child who is at least 16 years of age and when the child has consented to processing
	COPPA imposes certain requirements on operators of websites or online services aimed at children younger than 13 years and on those that have actual knowledge that they are collecting personal information online from a child under 13	For children under 16 years old “such processing shall be lawful only if and to the extent that consent is given or authorised by the holder of parental responsibility over the child” (article 8(1))

Several states have enacted comprehensive privacy laws, but these are often general rather than focused on reproductive and sexual health data.^20^ For example, the Colorado Privacy Act protects personal data of Colorado residents by granting them additional rights, such as the right to delete personal data and the right to know whether their personal data are being collected.^21^ By contrast, amendments to California’s Confidentiality of Medical Information Act promise heightened privacy protections specifically for reproductive and sexual health information on mobile applications and internet websites.^22^ ^23^ ^24^

In the EU, privacy is primarily governed by the General Data Protection Regulation (GDPR), which is much more comprehensive and stringent than state privacy laws in the US.^25^ The GDPR does not specifically regulate femtech,^26^ but article 9(1) contains a general ban on processing special categories of data, including data concerning health or a person’s sex life or sexual orientation, without the user’s consent or another clearly defined justification (article 9(2)). Femtech data may be considered as a special category of personal data and receive heightened protection because it falls under the categories of data concerning health (eg, period tracking data) or data on a person’s sex life or sexual orientation.

Both the US and EU data privacy frameworks provide protection to children’s personal data when using online services and apps aimed at children, emphasising the need to obtain parental consent or the child’s consent (table 1). Parallels can be drawn between femtech and apps for children since both technologies collect sensitive user generated data that risk being improperly disclosed. The provisions used for children therefore provide a framework that can be adapted to develop distinct safeguards that offer heightened privacy and security protections for increasingly vulnerable femtech data.

### Bias

Although bias is not unique to femtech, women often experience disproportionate harms from biometric technologies.^27^ These bias concerns apply both to consumer apps and to products aimed at supporting clinicians. Research suggests that many femtech applications are skewed towards western, educated, industrialised, and wealthy populations, highlighting the need to examine how apps may sometimes overlook other cultural contexts such as class, gender, social capital, digital access, digital literacy, language, and regional locations.^28^ For instance, in India, where reproduction is tied to family expectations and patriarchal norms, femtech may not always foster personal empowerment and may instead risk reinforcing social control and surveillance.^28^ Bias could result from algorithms being trained on data from one population and then applied to another, or simply that the apps are designed for a particular use and context with little thought given to how they might be used elsewhere. This is of particular concern for regulators in the global south.

Additionally, studies evaluating ethical concerns around the use of algorithmically driven period and fertility trackers highlight the challenge of making cycle predictions based on misguided evidence. Conclusions drawn from the collected data may not be universally applicable, particulary if the process of collecting data incorporates societal norms and pre-existing values of the people who designed the app, thus perpetuating biases in the results they predict.^29^ An example of inaccuracies arising from algorithmic bias is apps that predict a user’s fertile window based on the assumption that the user has a textbook 28 day cycle with ovulation occurring on day 14.^30^ This widely held assumption has been challenged more recently by data showing that ovulation timing and cycle length vary widely among Asian and Latina women, and are significantly influenced by factors such as developmental conditions, dietary practices, and levels of wealth.^29^

## Responsible use

Increasingly, healthcare providers are relying on femtech data to record patients’ menstrual and gynaecological pain and related symptoms, and some even recommend specific apps for tracking these conditions.^31^ Of 386 resident doctors surveyed in 19 California obstetrics and gynaecology programmes, 93% used specialty related apps in clinical settings, while only 53% respondents recommended apps to patients.^32^

Growing evidence also shows the risks of relying on femtech data for birth control, with the traditional rhythm method having a typical failure rate of 24%.^33^ For example, Natural Cycles, which became the first birth control app to be certified as a contraceptive method in Europe, was reported to Swedish authorities when a hospital found 37 cases of unwanted pregnancies among women who relied on the app for contraception.^33^ After investigation, Swedish regulators concluded that the number of pregnancies among users of Natural Cycles fell within the US Food and Drug Administration’s reviewed “typical use” failure rate, but requested that the company clarify that risk within the app, which Natural Cycles did.^34^ The investigation was closed in September 2018 with no further action required. 

With use of such products becoming more widespread, it is important to consider how to protect users privacy and ensure the value of apps is not undermined by bias.

### Heightened privacy protections

Healthcare providers can have an important role in strengthening femtech data privacy protections by recommending only those apps or wearables that meet established standards for clinical safety, data protection, technical security, interoperability, usability, and accessibility. A notable example is the UK’s digital technology assessment criteria,^35^ which support compliance for commissioning any digital health technologies for formal use across NHS and social care services.^35^ Similarly, use of applications in Germany can be reimbursed only if they meet robust criteria, which requires the inclusion of the digital health application in an official register maintained by the German medical regulatory body and a prescription from the treating medical practitioner or health insurer approval.^36^

Integrating data from femtech apps into healthcare systems, particularly electronic health records, has the potential to improve women’s reproductive and sexual health screening. Therefore, independent agencies or medical associations in other jurisdictions should also develop guidelines and best practices that providers can reference before prescribing apps. Such criteria must take into account data processing and privacy policies of femtech apps to help providers make informed recommendations and protect patients in clinical practice and care delivery. If providers recommend the use of an app to collect and store patient information or as a diagnostic tool, the obligation to keep the information confidential and secure should also extend to the provider, and not just oblige the app controller to respect data privacy.^35^

Federal healthcare agencies and departments in other jurisdictions should also develop frameworks that clarify the roles, responsibilities, and obligations of healthcare stakeholders, such as clinicians, when patients and providers incorporate femtech into care. Countries with patchwork provisions should take inspiration from the EU GDPR, which strives for comprehensiveness and consistency across data types. Another aspect of the GDPR that should be emulated is its strict emphasis on the rights of the data subject. These include the right to information about whether personal data have been collected and from where; rights of access, rectification, erasure, and restriction of processing; and the right to data portability.^37^ In addition, the GDPR’s risk based framework, which allows for heightened protections for special categories of data, such as health, is valuable.

### Addressing bias

Biases are perpetuated when femtech apps fail to account for the lived realities of each individual and reinforce stereotypes. One effective way to mitigate bias in femtech is for app developers to ensure that the training data for AI models is representative of all target populations, with consideration for their identities (eg, demographic data such as ethnicity, religion, and gender), including the specifics of their menstrual cycle, such as regularity, symptoms, and associated morbidities. By addressing the risk that technologies can reinforce exclusion and marginalisation of under-represented groups, femtech developers can design a more thoughtful user experience centered on representation and inclusion, resulting in more accurate reflections of fertility and ovulation outcomes.^29^

Regulatory agencies governing artificial intelligence (AI) should develop “AI facts labels”^38^—modelled on the standard nutrition facts labels—as well as a “front-of-package” AI label giving an easy to understand summary of the way a device or application works.^38^ These tools would improve user literacy and will be especially critical in the context of period and fertility trackers, as they can help users better interpret their results and make informed decisions about the technologies they use.^38^ For example, QuantX, machine learning based software that analyses magnetic resonance imaging (MRI) data to help radiologists detect breast cancer, did not report sex or ethnicity breakdowns of its dataset. Omission of this information, which is important for radiologists, fails to acknowledge that breast tissue density can vary across populations, such as people of African versus European ancestry, and may influence screening outcomes and diagnostic accuracy.^38^ AI fact labels may be especially important in countries with populations that differ significantly from that of the US and EU, where many of these algorithms have been trained.

## Practical implications

Integrating femtech into clinical practice may present challenges. In healthcare systems where providers are already overburdened by rapid technological advances^39^ (eg, AI in clinical decision making), staying abreast of changing privacy policies or innovations in femtech could further strain provider capacity. Moreover, since femtech transcends borders, a globally used app could be subject to different regulatory frameworks depending on where the user is located or data processed, making regulation complex.

Incorporating femtech into clinical practice could also result in excessive surveillance of routine activities such as mandatory reporting of menstruation data to monitor which individuals become pregnant and seek terminations. This may stifle innovation if overly stringent privacy safeguards prohibit data collection rather than preventing its misuse.^40^ Furthermore, overanonymising femtech data could reduce the value and accuracy of the information available and affect how healthcare providers screen, diagnose, and treat patients.

On the other hand, an overemphasis on privacy could hinder valuable health data collection, which is key to developing algorithms that are more accurate and less biased, and trained on a demographically reflective dataset.^40^ Therefore, a framework that balances privacy while addressing bias is critical.

**Key messages**

- Femtech is increasingly used for an array of female health needs such as maternal, menstrual, and sexual health
- Shifts in policy and social undercurrents have made femtech data more vulnerable, raising legal and ethical concerns around data privacy and bias
- Heightened privacy and bias safeguards are necessary to avoid improper use and disclosure of data
- Clinicians should be aware of these risks of femtech, and use and recommend only products that have been shown to properly address them

## Notes

Contributors and sources: SR is an attorney whose research focuses on digital health innovation, regulatory compliance, and how technology can expand access to care. SG is an expert on the ethical and legal challenges of AI in healthcare. EB is a physician and health policy researcher focused on the integration of technology into clinical care delivery. CSh is an expert in digital health, reproductive health, and emerging technologies. SR drafted the initial manuscript. All authors revised and edited the manuscript and approved the final version. CS is the guarantor.

Provenance and peer review: Commissioned; externally peer reviewed.

This article is part of a collection developed in partnership with the Gates Foundation. Open access fees were funded by the Gates Foundation. *The BMJ* commissioned, peer reviewed, edited, and made the decision to publish. Smruti Patel and Jocalyn Clark were the lead editors for *The BMJ*.
